# Assessment of compensated advanced chronic liver disease based on serum bile acids in chronic hepatitis B patients

**DOI:** 10.1038/s41598-023-39977-8

**Published:** 2023-08-08

**Authors:** Fei Chen, Yaning Yao, Zhen Li, Long Deng, Ruiling He

**Affiliations:** 1 Department of Ultrasound, The First Hospital of Lanzhou University, Lanzhou, 730000 China; 2 Department of Ultrasound, Donggang Branch, The First Hospital of Lanzhou University, Lanzhou, 730000 China

**Keywords:** Hepatitis, Liver cirrhosis, Liver fibrosis

## Abstract

Patients with chronic liver disease progressed to compensated advanced chronic liver disease (cACLD), the risk of liver-related decompensation increased significantly. This study aimed to develop prediction model based on individual bile acid (BA) profiles to identify cACLD. This study prospectively recruited 159 patients with hepatitis B virus (HBV) infection and 60 healthy volunteers undergoing liver stiffness measurement (LSM). With the value of LSM, patients were categorized as three groups: F1 [LSM ≤ 7.0 kilopascals (kPa)], F2 (7.1 < LSM ≤ 8.0 kPa), and cACLD group (LSM ≥ 8.1 kPa). Random forest (RF) and support vector machine (SVM) were applied to develop two classification models to distinguish patients with different degrees of fibrosis. The content of individual BA in the serum increased significantly with the degree of fibrosis, especially glycine-conjugated BA and taurine-conjugated BA. The Marco-Precise, Marco-Recall, and Marco-F1 score of the optimized RF model were all 0.82. For the optimized SVM model, corresponding score were 0.86, 0.84, and 0.85, respectively. RF and SVM models were applied to identify individual BA features that successfully distinguish patients with cACLD caused by HBV. This study provides a new tool for identifying cACLD that can enable clinicians to better manage patients with chronic liver disease.

## Introduction

Hepatitis B virus (HBV) infection is a major public health problem worldwide. The World Health Organization estimated that 257 million people were living with chronic HBV infection in the worldwide, making these patients at a high risk of developing cirrhosis, hepatic decompensation, and hepatocellular carcinoma^1^. China has the world’s largest burden of HBV infection. It is estimated that there are about 70 million HBsAg carriers (5–6% prevalence) and 20–30 million people with chronic hepatitis B in China^2^. Liver biopsy is the gold standard for the assessment of liver fibrosis and cirrhosis in patients with chronic liver disease. However, it is an invasive procedure that may be complicated by pain, liver parenchyma injury and hemorrhage^3^. The current indication for liver biopsy is mainly to determine the cause of liver disease in selected cases, and not to stage fibrosis. Some non-invasive methods are commonly used to assess hepatic fibrosis and cirrhosis, with suggested cutoff values being applied to guide clinical decision making^4–6^.

Since it is usually impossible to distinguish between severe fibrosis and cirrhosis in asymptomatic patients on clinical grounds, the Baveno VI consensus proposed the term “compensated advanced chronic liver disease (cACLD)” to better reflect that the spectrum of the two is a continuum^7^. A pragmatic definition of cACLD based on liver stiffness measurement (LSM) is aimed at stratifying the risk of decompensation, irrespective of histological stage or the ability of LSM to identify these stages^8^. Currently, LSM by transient elastography (TE) is the most widely used non-invasive method for assessing cACLD clinically. The renewing Baveno VII consensus suggested that patients with LSM-TE < 10 kilopascals (kPa) in the absence of other known clinical/imaging signs are sufficient to rule out cACLD^8^. Compared with TE, on the basis of conventional ultrasound images, two-dimensional shear wave elastography (2D-SWE) uses acoustic radiation force to generate shear waves, and can also form color coded images with different stiffness in the sampling frame, so as to effectively avoid non target structures and obtain more reliable tissue stiffness values^9,10^. Many studies have shown that the performance of 2D-SWE is equivalent to or even better than that of TE in assessing liver fibrosis and cirrhosis^11–13^.

Recently, bile acid (BA), endogenous compounds that undergo efficient enterohepatic circulation, have been confirmed as an important factor in the pathophysiology of the dynamic component of portal hypertension in both animal models and in humans^14,15^^.^ Therefore, the aim of our study was to develop prediction model based on individual BA profiles of serum samples to identify cACLD in patients with HBV infection.

## Methods

### Patient selection

**Figure 1:**
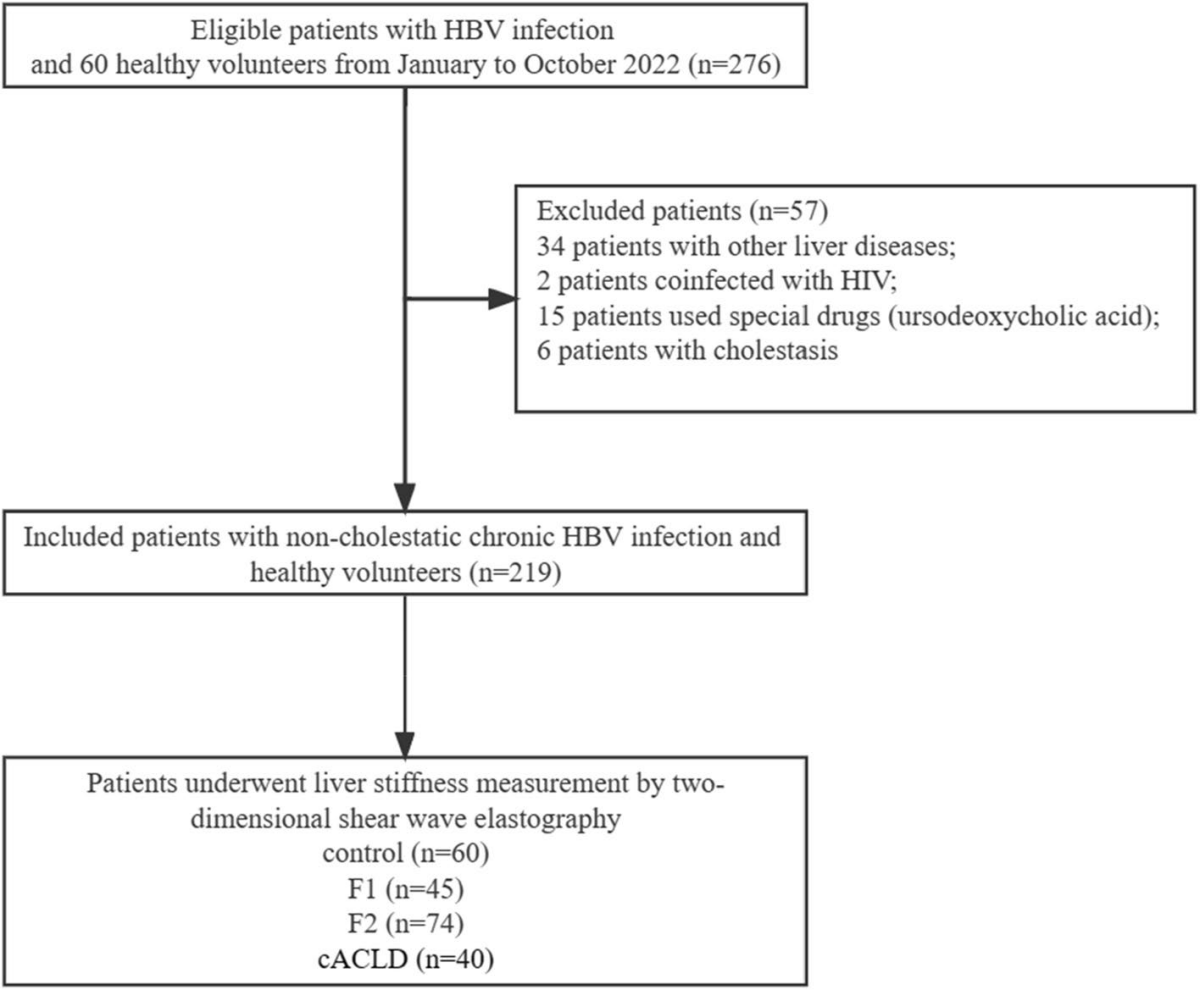
The flow chat of the study population. *HBV* hepatitis B virus, *HIV* human immunodeficiency virus, *cACLD* compensated advanced chronic liver disease.

This study was approved by The First Hospital of Lanzhou University Ethics Committee (approval number: LDYYLL2022-111). Data were collected from January to October 2022. Figure 1 shows the flow chat of the study population. We prospectively selected patients who met the following criteria: age between 18 and 75 years; positive serum hepatitis B surface antigen (HBsAg) for at least 6 months; accepted examination of LSM by 2D-SWE. Informed consent was obtained from all the study participants. Exclusion criteria were as follows: patients who used drugs against BA accumulation, such as ursodeoxycholic acid; hepatitis A, C, D, E viral infections; autoimmune liver disease; non-alcoholic fatty liver disease; alcoholic liver disease; drug-induced hepatitis; hepatocellular carcinoma; history of endocrine diseases and cholestasis. Cholestasis was defined as serum alkaline phosphatase (ALP) level > 1.5 ULN and gamma-glutamyl transpeptidase (GGT) level > 3 ULN. The ULNs of ALP and GGT were 125 U/L and 69 U/L, respectively.

In addition, 60 healthy volunteers were recruited as control with older than 18 years. They were all volunteers who had no substantial past medical history of chronic liver disease. They had no substantial alcohol intake (< 30 g/day for man, < 20 g/day for women) and had negative hepatitis B, hepatitis C, and human immunodeficiency virus.

All methods were performed in accordance with the relevant guidelines and regulations.

### Laboratory tests

Blood samples were collected from all subjects in a fasting state in the early morning. Biochemical parameters, including serum aspartate aminotransferase (AST); alanine aminotransferase (ALT); ALP; GGT; total bilirubin (TBIL); direct bilirubin (DBIL); indirect bilirubin (IBIL) and total bile acids (TBA) were determined using a fully automated biochemical analyzer (Olympus AU400, Japan).

### Two-dimensional shear wave elastography

All subjects underwent LSM based on 2D-SWE by a single professionally trained operator in fasting patients (fasted for > 6 h). An Aixplorer^®^ ultrasound system (SuperSonic Imagine, SSI, France) with an abdominal 3.5-MHz curved array probe was used. LSM was measured in the right lobe using the 7th to 9th rib intercostal approach, with the right arm in maximum abduction. The region of interest, size 4 cm × 3 cm and fan-shaped, was placed in an area of parenchyma free of large vessels and bile ducts, avoiding noisy areas from rib shadowing. Start the 2D-SWE measurement, place the Q-BOX at least 1 cm and no more than 6 cm away from the liver capsule, and the diameter is not less than 1.5 cm. The value of LSM was depicted in kPa. The latest European Federation of Societies for Ultrasound in Medicine and Biology (EFSUMB) guidelines were followed, the stiffness of the liver was measured five times in each case, and the median values were recorded^9^. The reliable value of LSM was defined as the stability index of image ≥ 80% and interquartile range/median ratio < 30%^9^.

The value of LSM ≤ 7.0 kPa was defined as F1, 7.1–8.0 kPa F2 and ≥ 8.1 kPa equal to or higher than F3. Patients with cACLD were considered to have the same or higher stage of liver fibrosis as F3^16^.

### Sample preparation and high performance liquid chromatography (HPLC) analysis

We measured BA in the serum using previously described Liquid Chromatograph Mass Spectrometer method^17^. Simple protein precipitation using methanol was used to prepare the serum samples. Briefly, 200 μL methanol was added to 100 μL of serum spiked with 100 μL of ISs (*d*_4_-chenodeoxycholic acid and Nor-desoxycholic acid). Subsequently, all the mixtures were vortexed for 1 min and centrifuged at 13,000×*g* for 5 min. The supernatant was aspirated for further analysis. An Agilent 1260 Infinity HPLC coupled with an Agilent 6460 triple-quadrupole mass spectrometer equipped with an electrospray ionization interface was used for the analysis of serum. Chromatographic resolution was performed on an Agilent HC-C18 column (4.6 mm × 250 mm, 5-μm particles), guarded by an Agilent Eclipse XDB-C18 4.6 mm × 12.5 mm analytical guard column (Agilent Technologies, USA). The mobile phase consisted of methanol (solvent B) and 7.5 mM ammonium acetate containing 0.1% ammonium hydroxide (solvent A, deionized water), pH 7.5, at a total flow of 1 mL/min, and post column splitting (1:4) was applied to give optimal interface flow rates (0.2 mL/min) for MS detection.

### Principal component analysis (PCA)

Principal component analysis (PCA) was performed using the prcomp (version 4.0.2) package to visualize the distribution of individual BA in different classes.

### Random forest (RF)

RF was introduced as a classifier owing to its attractive characteristics, including the need for few tunable parameters, automatic handling of missing data, and insensitivity to overfitting^18^. By using all the descriptors in the training set to build an RF classification model based on the cross-validation method, the importance of each descriptor with respect to prediction ability was determined. Subsequently, the order of importance for all descriptors was obtained. The resulting model was implemented in the statistical language R based on STATISTICA 10.0 with the default settings. The BA profiles of the control, F1, F2 and cACLD group were randomly divided into training and test sets at a 4:1 ratio, respectively 175 and 44 patients. There are two important parameters, ntree (the number of trees) and mtry (the number of features to split on each node), that must be optimized. In this study, to obtain the optimal model, the value of ntree was tuned from 1 to 219 with a step of 100. Meanwhile, the value of mtry was tuned from 1 to 50 with a step of 1 in each tuning step of ntree.

### Support vector machine (SVM)

The SVM method is a novel small-sample learning method, which can be used to deal with highly nonlinear regression and classification problems^19^. In brief, it is a supervised learning method that predicts the corresponding category of the new training sample by learning the category of the known sample and judging the relationship between the sample and the category. Similarly, all subjects were randomly divided into training and test sets with the ratio of 4:1. SVM was developed in a training set of 175 patients and tested in a validation set of 44 patients.

### Model validation

The performance of the classification models was evaluated using the following metrics: Marco-Precise, Marco-Recall, Marco-F1 score, total accuracy, and Kappa coefficient.

### Statistical analysis

Continuous variables were reported as median with interquartile range or mean with standard deviation. Categorical data, presented as number and frequencies (%). Differences among groups were analyzed by one-way analysis of variance with Dunnet’s multiple comparison test or Mann–Whitney test using SPSS 25.0.02 (IBM, New York, U.S.). The difference was considered statistically significant when *p* < 0.05. RF and SVM data acquisition and quantification were performed using STATISTICA 10.0.

## Results

### Characteristics of the study population

A total of 159 patients with HBV infection and 60 healthy volunteers from the First Hospital of Lanzhou University between January 2022 to October 2022 were included in the final analysis. The characteristics of the study population are summarized in Table 1. The mean age was (44.7 ± 11.6) years and 62% were males. F2 group was present in 74 patients, accounting for the largest proportion (33.8%). This was followed by control (27.4%, 60/219) and F1 group (20.5%, 45/219). cACLD was found in 18.3% (40/219) of patients. There was no significant difference in biochemical indexes between F1 and control group (*p* > 0.05). Patients with F2 and cACLD group had significantly elevated levels of AST, TBIL, DBIL, IBIL, and TBA compared to those in the control group (*p* < 0.05). In addition, the subjects with cACLD exhibited notable increase in ALT, ALP, and GGT comparing with the control (*p* < 0.05).

**Table 1:** Basic characteristics of the included patients.

	Control (n = 60)	F1 (n = 45)	F2 (n = 74)	Cacld (n = 40)
Age (years), mean (SD)	44 ± 11	41 ± 10	46 ± 11	48 ± 10
Male, n (%)	66.7%	73.3%	63.0%	60.0%
AST (U/L), median (IQR)	26.0 (11.8)	27.0 (9.2)	33.3 (18.6)**	43.0 (29)**
ALT (U/L), median (IQR)	25.5 (17.3)	29.5 (17.8)	32.9 (21.8)	32.3 (31.1)
ALP (U/L), mean (SD)	90.1 ± 25.3	86.5 ± 27.4	93.9 ± 27.5	105.2 ± 30.6*
GGT (U/L), mean (SD)	30.9 ± 12.6	28.8 ± 17.8	32.9 ± 18.8**	40.4 ± 27.1**
TBIL (μmol/L), median (IQR)	15.5 (8.2)	16.9 (6.7)	17.9 (9.8)	23.6 (15.6)**
DBIL (μmol/L), median (IQR)	7.1 (3.0)	7.8 (2.8)	8.4 (3.4)	11.0 (5.2)**
IBIL (μmol/L), median (IQR)	8.8 (4.6)	9.6 (4.3)	10.6 (5.3)	13.4 (5.8)**
TBA, mean (SD)	11.9 ± 8.7	15.5 ± 5.7	27.4 ± 15.4*	48.7 ± 17.2**

*SD* standard deviation, *IQR* interquartile range, *cACLD* compensated advanced chronic liver disease, *AST* aspartate aminotransferase, *ALT* alanine aminotransferase, *ALP* alkaline phosphatase, *GGT* γ-glutamyl transpeptidase, *TBIL* total bilirubin, *DBIL* direct bilirubin, *IBIL* indirect bilirubin, *TBA* total bile acids.

*p < 0.05, **p < 0.01 when compared to patients with control group.

### PCA

**Figure 2:**
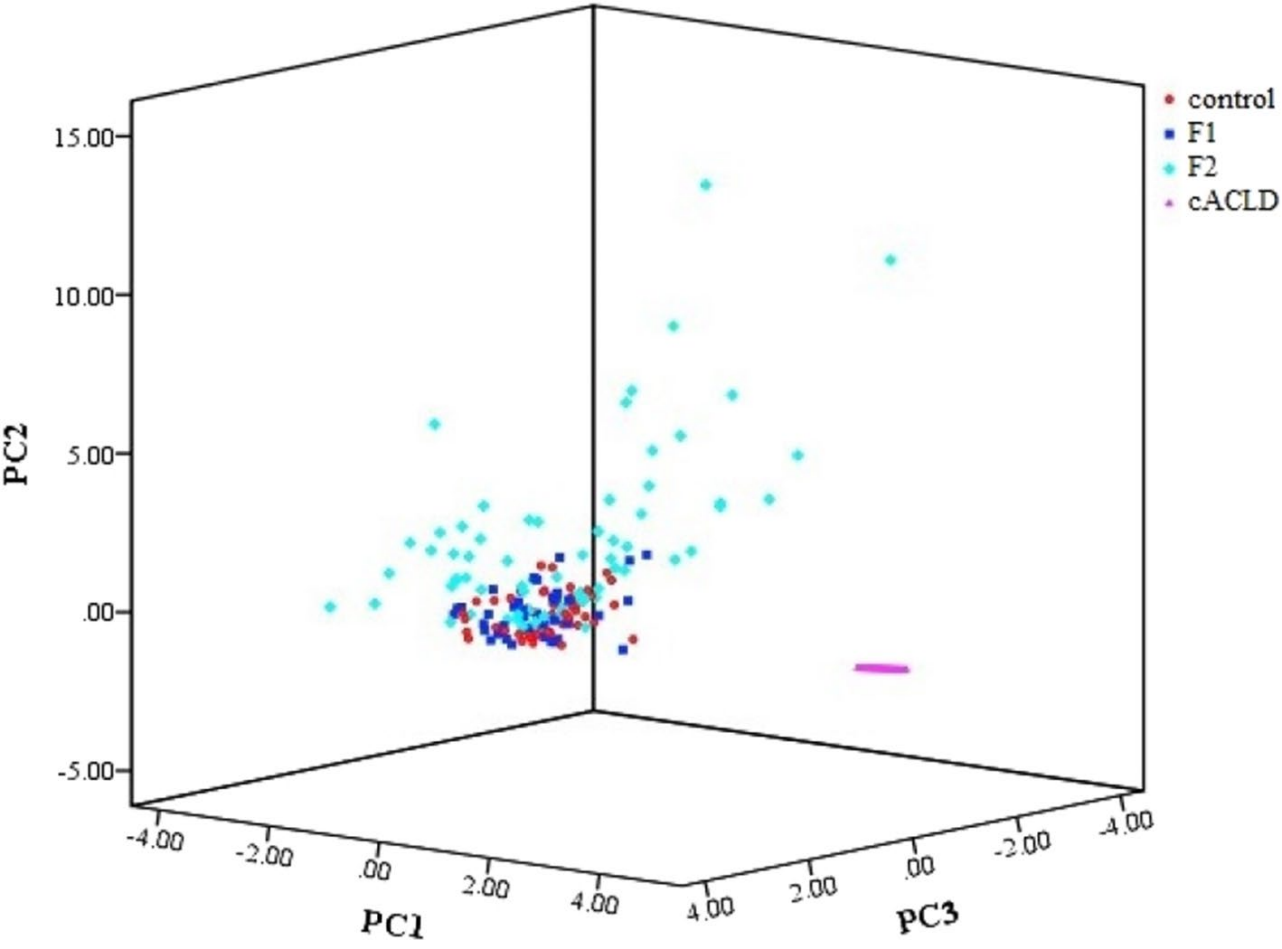
Principal component analysis in the control, F1, F2 and cACLD group. cACLD, compensated advanced chronic liver disease. Control (red); F1 (blue); F2 (green); cACLD (pink).

PCA was performed to visualize the distributions of individual BA profiles in different degrees of liver fibrosis. As shown in Fig. 2, the BA of cACLD (pink) patients yielded higher PC1, PC2 and PC3 values, and only a few outliers were evident between control, F1, and F2. Therefore, the PCA method yielded a clear separation of cACLD patients from chronic liver disease caused by HBV using the individual BA.

### The alteration of serum bile acids in different liver fibrosis stage

**Figure 3:**
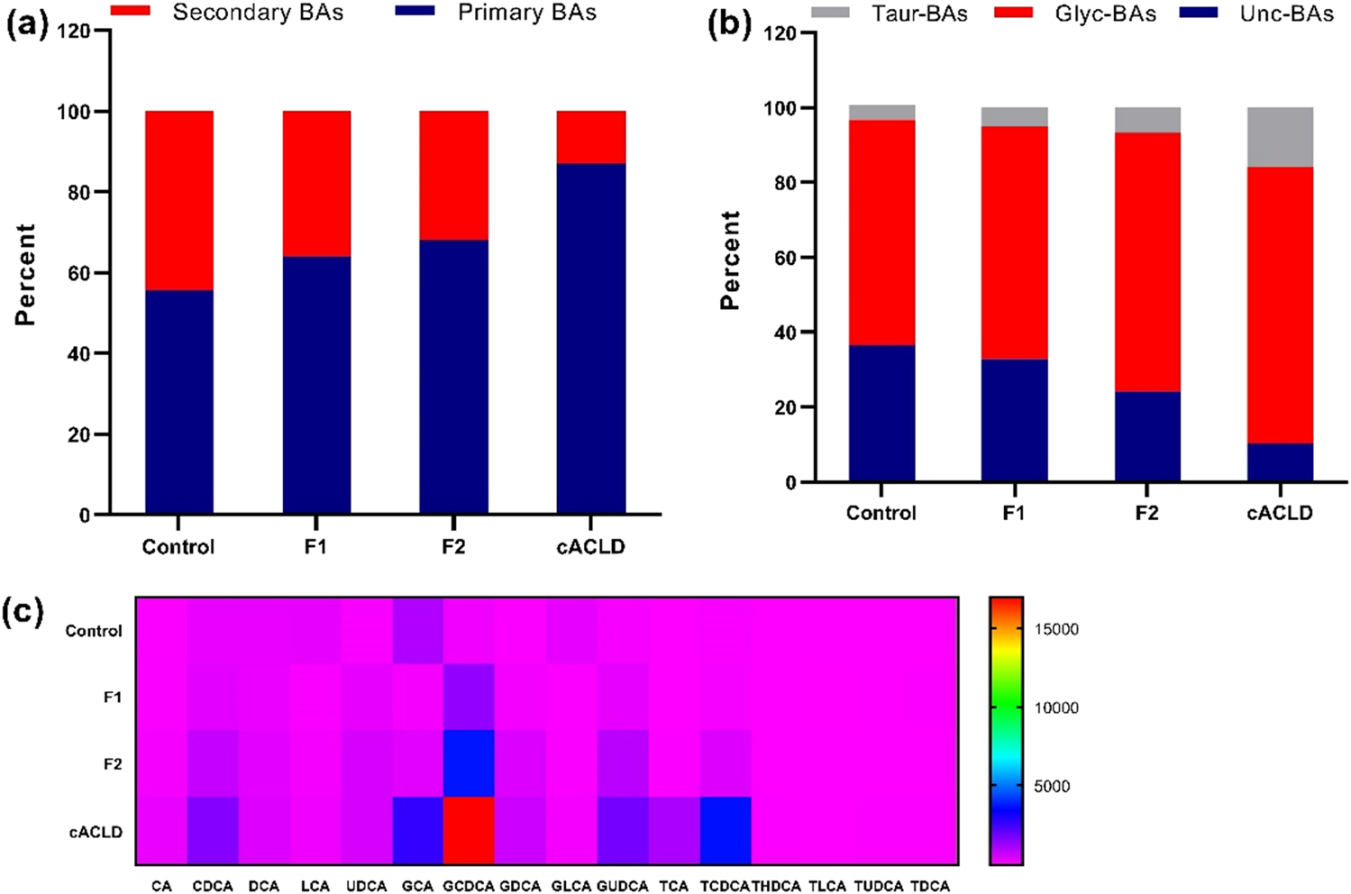
The alteration of serum bile acids in the control, F1, F2 and cACLD group. (**a**) Stack bar plot representing proportion of unconjugated (Unc-BAs), glycine conjugated (Glyc-BAs) and taurine conjugated (Taur-BAs) bile acids. (**b**) Stack bar plot representing proportion of primary bile acids and secondary bile acids; (**c**) heat map display the spectrum of bile acids profile across different fibrosis stage.

Compared to control, the subjects with F1, F2, and cACLD exhibited an increase in total serum primary BA, while the proportion of total secondary BA decreased (Fig. 3a). In addition, patients with F1, F2, and cACLD exhibited significant increases in glycine-conjugated BA and taurine-conjugated BA compared with the control (Fig. 3b). The heat map displays the spectrum of the bile acid profiles across different fibrosis stage (Fig. 3c). Compared to control, there was a significant increase in the percentage of glycine-conjugated BA in F1 (2%), F2 (9%), and cACLD patients (14%). Taurine-conjugated BA exhibited a higher percentage in F1 (1.1%) and F2 patients (2.2%) and a significantly higher percentage in cACLD patients (12%). Moreover, the percent of unconjugated BA was 36% for the control, 33% for F2, 24% for F3, and 10% for cACLD group.

### The change of serum individual bile acids in different liver fibrosis stage

**Figure 4:**
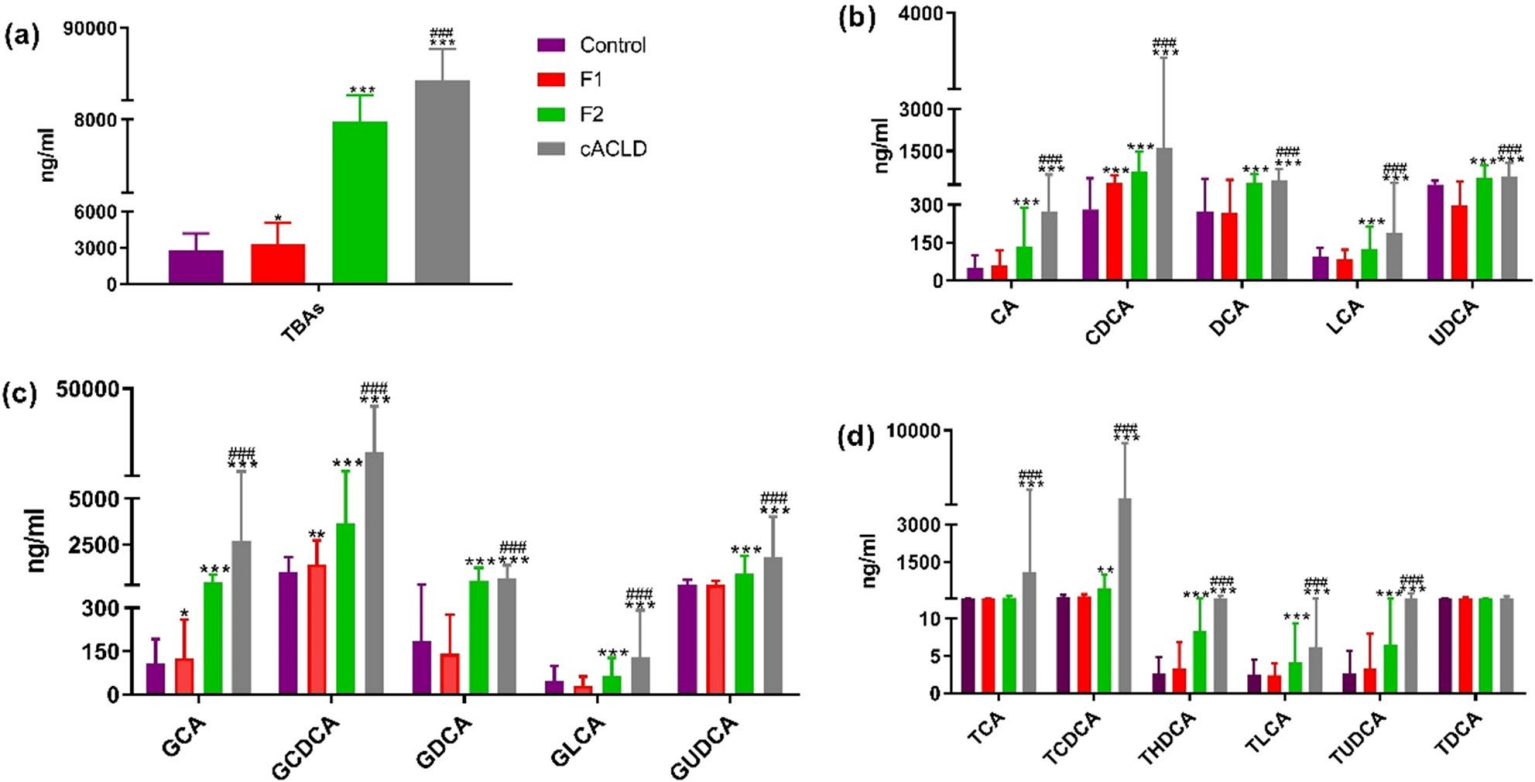
The change of TBAs, unconjugated, glycine-conjugated and taurine-conjugated bile acids of (mean ± SD) in the control, F1, F2 and cACLD group. TBAs: the sum of unconjugated, glycine-conjugated and taurine-conjugated bile acids content in the serum. *cACLD* compensated advanced chronic liver disease. Compared with the control, *p < 0.05,**p < 0.01, ***p < 0.001; Compared with F2 group, ^###^p < 0.001.

Compared to the control, the sum of unconjugated, glycine-conjugated, and taurine-conjugated BA contents in the serum was significantly increased in F1, F2, and cACLD patients (Fig. 4a). Compared to the control, F1 patients showed a significant increase in the content of CDCA (*p* < 0.001), GCA (*p* < 0.05), and GCDCA (*p* < 0.01) (Fig. 4b, c), but there was no significant difference in other individual BA. Unconjugated BA such as CA, CDCA, DCA, LCA, and UDCA in the serum of patients with F2 were significantly increased (*p* < 0.001), and glycine-conjugated BA such as GCA, GCDCA, GDCA, GLCA, and GUDCA were also significantly increased (*p* < 0.001) in those with F2 (Fig. 4c). Furthermore, TCDCA, THDCA, TLCA, and TUDCA were significantly increased (*p* < 0.001) in patients with F2 (Fig. 4d). All individual BA were significantly increased in the serum of cACLD patients, except for TDCA.

### Classification performance of RF and SVM

**Figure 5:**
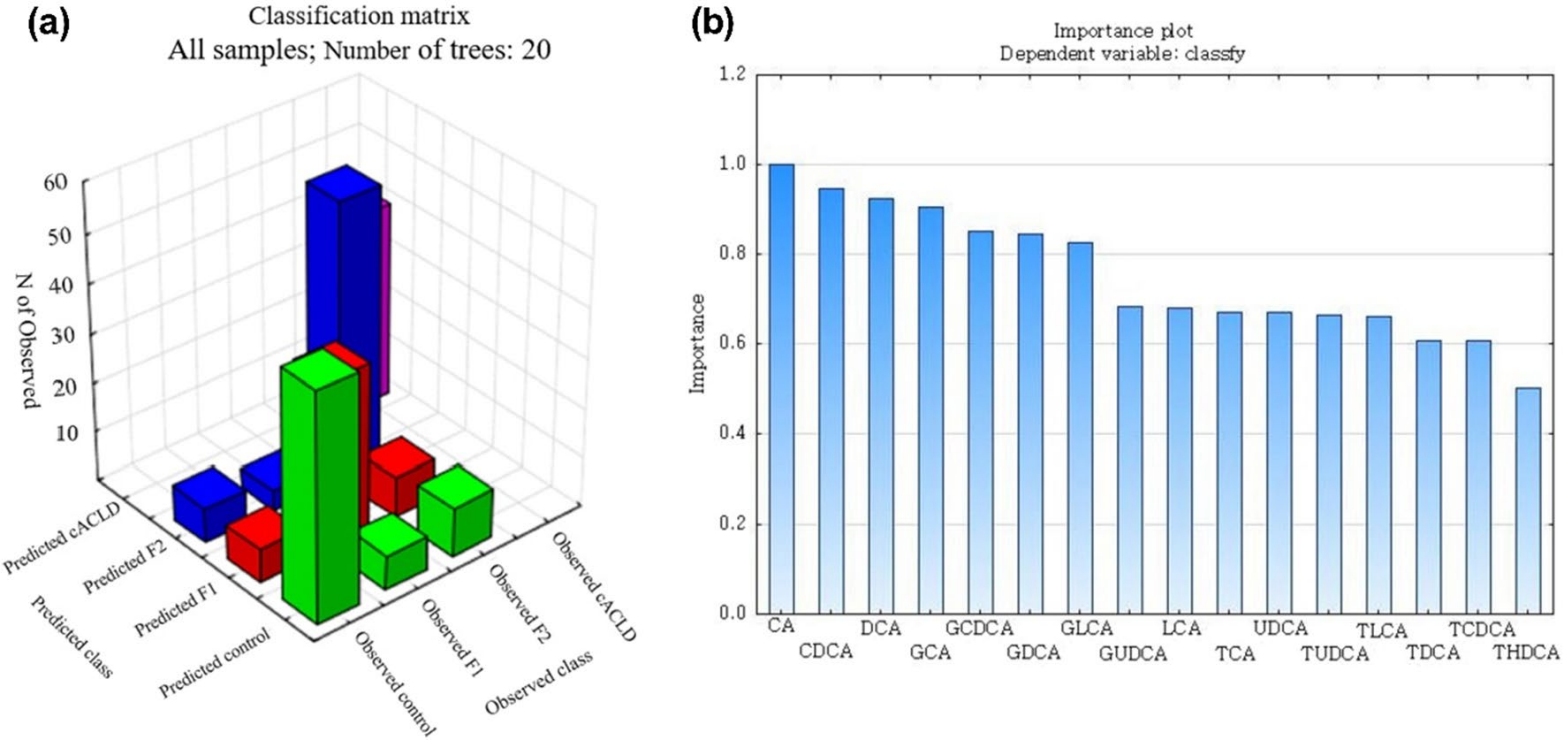
The performance of build Random Forest (RF) model. (**a**) Classification matrix of all samples, number of trees: 20; (**b**) importance plot of individual bile acids.

The number of decision trees was set to 20, and the maximum tree size was set to 15 based on the results of the parameter tuning tests. A summary of the RF response of classification is shown in Fig. 5a. For the RF method, a regression algorithm based on importance ranking was used to extract the features of the impact factors, select the optimal feature variable set, and achieve the goal of dimension reduction. The SVM model was optimized using tenfold cross-validation, and the selected samples were trained and predicted using the SVM model. The Marco-Precise, Marco-Recall, Marco-F1 score, kappa coefficient, and accuracy of the optimized RF model were 0.82, 0.82, 0.82, 0.74, and 0.81, respectively. The importance of 16 individual BA features was calculated using the RF method. The importance status is shown in Fig. 5b, showing that all the assigned individual BA features had the capacity to discriminate between different liver fibrosis stage. The CA, CDCA, DCA, GCA, and GCDCA features gained the highest importance. The Marco-Precise, Marco-Recall, Marco-F1 score, kappa coefficient, and accuracy of the optimized SVM model were 0.86, 0.84, 0.85, 0.76, and 0.82, respectively. The performances of the built RF and SVM models for identifying different liver fibrosis stage are shown in Table 2.

**Table 2:** Performance of the built random forest (RF) and support vector machine (SVM) models for identification different liver fibrosis stage.

Model	Marco-Precise	Marco-Recall	Marco-F1 score	Kappa coefficient	Accuracy
RF	0.82	0.82	0.82	0.74	0.81
SVM	0.86	0.84	0.85	0.76	0.82

Marco-F1 score: 2 × Marco-Precise × Marco-Recall/(Marco-Precise + Marco-Recall).

## Discussion

The degree of liver fibrosis in patients with chronic liver disease predicts the likelihood of developing liver-related morbidity and death^20^. When these patients progressed to cACLD, the risk of liver-related decompensation events and death increased significantly. Thus, assessment of cACLD is an essential part of the evaluation of chronic liver disease patients in order to prognosticate, stratify therapeutic and surveillance strategies^8^. Research showed that elevated serum BA concentrations have been shown to be a more sensitive test for the detection of liver cirrhosis than conventional liver function tests^14^. Therefore, this study developed two different models (RF and SVM) based on individual BA profiles of serum samples to recognize cACLD in patients with HBV infection.

In the present study we analysed the relationship between serum concentrations of individual BA and the degree of liver fibrosis in patients with chronic liver diseases. We found that PCA method using the individual BA can distinguish cACLD from chronic liver disease patients. Furthermore, the total serum primary BA increased while the proportion of total secondary BA decreased in subjects with the controlled, F1, F2, and cACLD group. Meanwhile, the glycine-conjugated BA and taurine-conjugated BA increased, while the unconjugated BA decreased. More importantly, conjugated BAs, including GCDCA and TCDCA, increased significantly in patients with cACLD. It seems to be consistent with the research of Žížalová et al., which found that GCDCA and TCDCA are significantly related to portal pressure in patients with cirrhosis^14^. Oehler showed that the synthesis of primary BA, CA and CDCA, was significantly increased by the strong induction of hCYP7A1 (the rate-limiting enzyme converting cholesterol to BA) in human liver chimeric mice infected with HBV^21^. Although the relative excess of CDCA over CA derivatives seem to be a common feature of liver cirrhosis as well as non-alcoholic fatty liver disease, the mechanism behind this remains somewhat enigmatic^22^. The Žížalová K’s study aimed to identify clinically significant portal hypertension in patients with cirrhosis through BA, while our study intended to identify cACLD in patients with chronic liver disease, which represent an important point for timely intervention to prevent further progression.

Furthermore, the RF model and SVM model derived from our current BA analysis showed good separation between different fibrosis stage, highlighting the diagnostic potential of this noninvasive analytical approach. Five serum BA, CA, CDCA, DCA, GCA, and GCDCA, gained the highest importance, suggesting that unconjugated and glycine-conjugated BA may be indicators of liver dysfunction in chronic hepatitis.

The limitation of the current study was that we were unable to compare noninvasive biomarkers with the gold standard of liver biopsy. In addition, the subjects included in present study are all patients with HBV. The results need to be verified in patients with other causes, such as hepatitis C virus, alcoholic, non-alcoholic fatty liver disease, autoimmune liver disease, etc.

In conclusion, RF and SVM models were applied to identify individual BA features that successfully distinguish patients with cACLD caused by HBV. This study provides a new tool for identifying cACLD patients that can enable clinicians to better manage patients with chronic liver disease.

## Data Availability

The authors confirm that the data supporting the findings of this study are available within the article.

## Abbreviations

TCA: Taurocholic acid
TCDCA: Taurochenodeoxycholic acid
TUDCA: Tauroursodeoxycholic acid
TDCA: Taurodeoxycholic acid
THDCA: Taurohyodexycholic acid
TLCA: Taurolithocholic acid
GUDCA: Glycoursodeoxycholic acid
GDCA: Glycodeoxycholic acid
GCDCA: Glycochenodeoxycholic acid
GLCA: Glycolithocholic acid
GCA: Glycocholic acid
CA: Cholic acid
UDCA: Ursodeoxycholic acid
DCA: Deoxycholic acid
CDCA: Chenodeoxycholic acid
LCA: Lithocholic acid
